# Characterization of the Baeyer–Villiger monooxygenase in the pathway of the bacterial pyrrolizidine alkaloids, legonmycins†

**DOI:** 10.1039/d4cb00186a

**Published:** 2024-09-30

**Authors:** Shan Wang, Fleurdeliz Maglangit, Qing Fang, Kwaku Kyeremeh, Hai Deng

**Affiliations:** a State Key Laboratory of Microbial Technology, Shandong University Qingdao 266237 China shan.wang@sdu.edu.cn; b Department of Chemistry, School of Natural and Computing Sciences, University of Aberdeen Aberdeen AB24 3UE UK h.deng@abdn.ac.uk; c Department of Biology and Environmental Science, College of Science, University of the Philippines Cebu Lahug Cebu City 6000 Philippines; d Marine and Plant Research Laboratory of Ghana, Department of Chemistry, University of Ghana P.O. Box LG56 Legon-Accra Ghana

## Abstract

The Baeyer–Villiger monooxygenase (BVMO), LgnC, plays a crucial role in the biosynthesis of bacterial pyrrolizidine alkaloids, legonmycins. It processes bicyclic indolizidine substrates generated from the coordinative action of two non-ribosomal peptide synthetases (LgnB and LgnD) and the standalone type II thioesterase-like enzyme (LgnA). It has been demonstrated that the enzyme selectively inserts molecular oxygen into the carbon–carbon bond adjacent to the carbonyl group in legonindolizidines to form bicyclic 1,3-oxazepine carbamate intermediates. After ring opening and contraction, the most advanced products, prelegonmycins, are formed. However, factors controlling the final hydroxylation step and how the enzyme handles the substrates have remained elusive. In this study, we show that the final hydroxylation at the activated carbon of the electron-rich pyrrole system is attributed to either spontaneous oxidation or the action of an endogenous redox reagent. Substrate docking on the structural model of LgnC combined with site-directed mutagenesis allows the identification of several key amino acids that are essential for substrate/intermediate binding and a mechanism of LgnC-catalysed transformation is proposed.

## Abstract 2

The Baeyer–Villiger monooxygenase (BVMO), LgnC, plays a crucial role in the biosynthesis of bacterial pyrrolizidine alkaloids, legonmycins.

## Graphical abstract

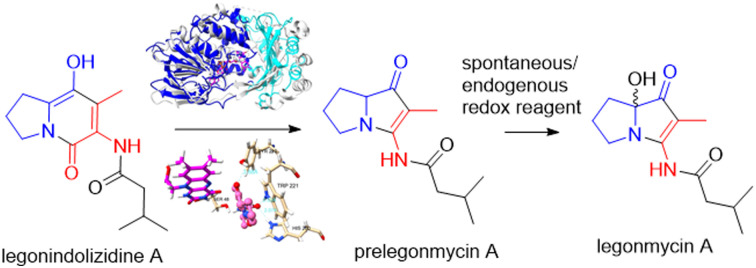

## Introduction

The Baeyer–Villiger (BV) oxidation is a valuable organic transformation that converts ketones and lactones into linear or cyclic esters, respectively, by introducing an oxygen atom, resulting in a carbonyl C–O linkage.^1^ In nature, a class of enzymes known as Baeyer–Villiger monooxygenases (BVMOs) also catalyse this reaction.^1–3^ These flavin-dependent enzymes facilitate the insertion of an oxygen atom from molecular oxygen. They do this precisely between a carbonylic carbon (Csp^2^) and the adjacent carbon (Csp^3^) of ketones and aldehydes. Moreover, BVMOs exhibit the ability to oxidize molecules containing heteroatoms, such as sulphides, amines, and boron compounds.^4,5^ Extensive studies have shown that these enzymes can oxidize a diverse array of substrates with remarkable chemo-, regio-, and enantioselectivity. As a result, they can create intriguing chemical moieties, including carbonates, twisted spiroketal functionalities, and dearomatized products, which are prevalent in various natural product biosynthetic pathways.^6–13^

**Fig. 1:**
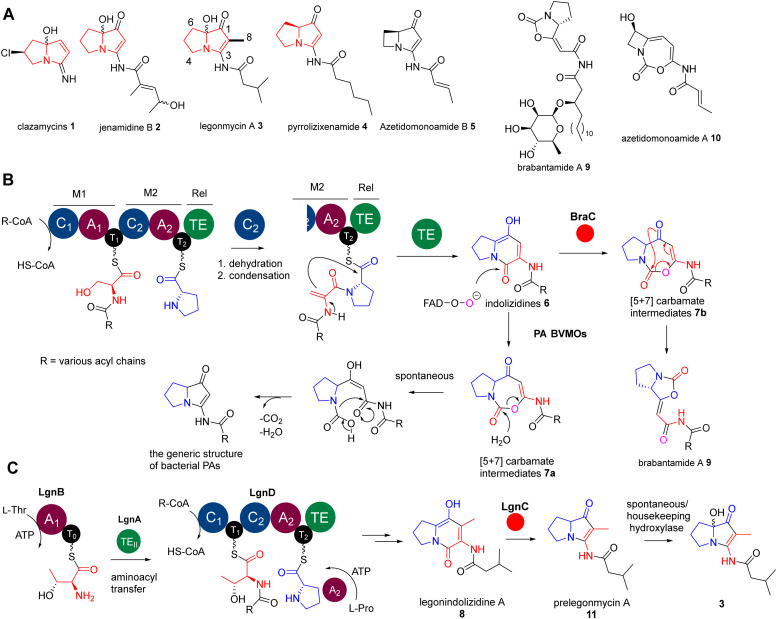
(A) Representative structures of naturally occurring bacterial pyrrolizidine alkaloids (PAs) and biosynthetically related lipocarbamates and cyclocarbamates. PAs 1–3 contain a hydroxyl group at the C7 position. (B) The biosynthesis of bacterial PAs and lipocarbamates only requires two essential enzymes, bimodular NRPS enzymes (C_1_–A_1_–T_1_–C_2_–A_2_–T_2_–Te) and diverged BVMOs, to provide these two distinct scaffolds. (C) A rather unusual pathway of legonmycins which rather require the presence of four enzymes, LgnA-D, to complete the biosynthesis. The atypical TEII enzyme domain, LgnA, is essential to catalyse the aminoacyl transfer between two T domains of two NRPSs, LgnB and LgnD.

Pyrrolizidine alkaloids (PAs) are a group of heterocyclic specialised metabolites, featured by two-fused 5-membered rings with a nitrogen atom at the bridgehead. While PAs have been mainly found as plant metabolites, less than 40 PAs have been discovered from bacterial origins, many of which exhibit potent anticancer and antimicrobial activities.^14^ The representatives include clazamycins 1,^15,16^ jenamidine B 2,^17^ legonmycins 3,^18^ pyrrolizixenamide 4^19^ and azetidomonoamide B^20,21^ (or azabicyclene) 5 (Fig. 1(A)). Some of these PAs (*i.e.*1–3) exist as isomers as they possess an achiral hydroxyl group at the C7 position on the carbon bridgehead of their bicyclic rings (Fig. 1(A)). It has been proposed that, although the hydroxylation at C7 may occur through enzymatic reactions in a stereospecific manner, the products undergo spontaneous racemization at neutral pH.^15,18^

Recent biosynthetic studies demonstrated that the biosynthesis of bacterial PAs starts with the production of bicyclic [5+6] indolizidine intermediates 6, resulting from the action of bimodular non-ribosomal peptide synthetases (NRPSs) in most bacterial PA pathways^18–22^ (Fig. 1(B)). These indolizidine intermediates are further modified by a group of pathway-specific BVMO enzymes, which catalyse a Baeyer–Villiger ring expansion from 6 to [5+7] 1,3-oxazepine carbamates 7^18^ (Fig. 1(B)). Subsequent non-enzymatic hydrolysis leads to ring opening and decarboxylative contraction, ultimately yielding [5+5] PA ring systems, as demonstrated in a recent report (Fig. 1(B)).^23^ In the case of legonmycins, their biosynthesis, however, requires four enzymes, where LgnB and LgnD are NRPSs, possessing domain arrangements of A_1_–T_0_ and C_1_–T_1_–C_2_–A_2_–T_2_–TE, respectively (Fig. 1(C)). It has been demonstrated that LgnA, an unusual type II thioesterase (TE_II_), catalyses the essential aminoacyl translocation from LgnB-T_0_ to LgnD-T_1_, facilitating downstream reactions^24^ (Fig. 1(C)). Biochemical analysis also showed that the LgnD-C_2_ domain, a member of unusual C domains,^25^ catalyses the essential dehydration reaction on LgnD-T_1_-tethered acyl-Thr to provide the corresponding acyl-dehydrobutyrine intermediates^24^ (Fig. 1(C)).

Interestingly, the pathways of bacterial PAs share the same bimodular NPRS architectures with the lipocyclocarbamates, brabantamides 9, to provide intermediates with identical [5+6] indolizidine ring systems 7b (Fig. 1(B)).^26,27^ However, a homologue of PA BVMOs, BraC, encoded in the corresponding biosynthetic gene cluster, catalyses a diverse ring re-arrangement of the [5+7] 1,3-oxazepine carbamates to provide 9 (Fig. 1(B)).^23^ In some cases, the [5+7] 1,3-oxazepine carbamates produced by these BVMOs could be trapped or further modified by other enzymes to form stable products, which are then released into the fermentation broth. This can be exemplified in clipibicyclenes 10,^28^ cyclocarbamides,^29^ legoncarbamate,^30^ and SB-315021^31^ (Fig. S1, ESI†), which were found to be potent antimicrobial agents. Recently, it has been shown that 10 is a potent covalent inhibitor of bacterial caseinolytic proteases (ClpPs),^28^ which are part of bacterial highly conserved proteolytic complexes that are, in many cases, essential for growth.

In this study, we report that the recombinant LgnC can efficiently mediate the biotransformation of legonindolizidine A 8 to prelegonmycin A 11 (Fig. 1(C)). However, the hydroxylation at the C7 position of 11 to form 3 in our *in vitro* assays likely results from spontaneous oxidations. Assays of legonindolizidine A or purified prelegonmycin A with *E. coli* whole cells where the recombinant LgnC was overexpressed along with the necessary cofactors, demonstrate that legonmycin A is efficiently produced, strongly indicating that the hydroxylation at the C7 position of 11 is mediated by an endogenous hydroxylase. Structural modelling of LgnC with its cofactor FAD and subsequent substrate docking analysis allow the identification of key amino acid residues in its putative active site. Site-directed mutagenesis (SDM) experiments coupled with bioinformatic analysis indicate that H200, Y219, and W221, which are highly conserved in PA BVMOs, play important roles in the BV biotransformation from 8 to 11. Finally, a mechanism of LgnC-mediated transformation is proposed.

## Results and discussion

It was demonstrated that the recombinant LgnC with a trigger factor (TF) tag was able to convert legonindolizidine A 8 to legonmycin A 3 (Fig. 1). Extracted ion chromatography (EIC) of our LC–MS analysis showed the presence of proposed intermediates during the biotransformation catalysed by LgnC. However, only a trace amount of legonmycin A 3 was observed.^18^

**Fig. 2:**
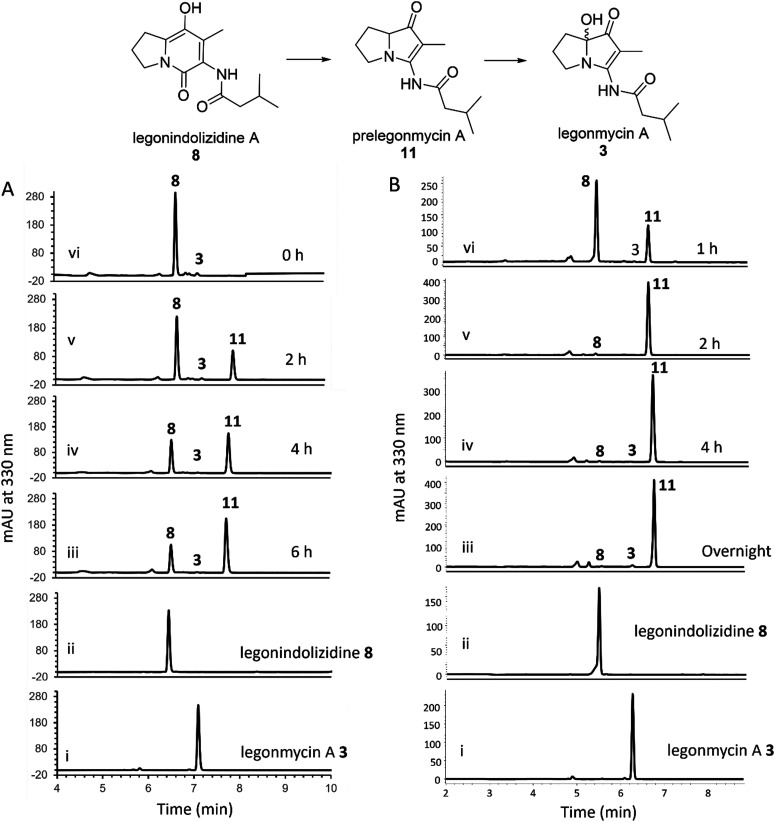
HPLC traces of the biotransformation from 8 to 3. (A) HPLC analysis of the conversion of 8 to 11 and 3 using purified recombinant LgnC-TF. Trace i. standard legonmycin A 3; ii. The standard 8; iii–vi. The time course assays showing the conversion of 8 to 11 and 3 (0 h, 2 h, 4 h, 6 h). (B) HPLC analysis of the conversion of 8 to 11 and 3 using cell-free extract of M1152-lgnC containing overexpressed recombinant His_6_-LgnC. Trace i. the standard of 3; ii. The standard of 8; iii–vi. The time course assay shows the conversion of 8 to 11 and 3 (1 h, 2 h, 4 h, overnight).

Efforts were then made to optimize the reaction conditions to improve the efficient biotransformation from 8 to 3. Incubation with the recombinant LgnC-TF in the presence of 8 (acquired from our previous studies^18^) together with FAD (0.1 mM), NADP^+^ (1 mM), and the NADPH recycling system^18^ has only resulted in a trace amount of 3 (by HPLC) (Fig. 2(A)) as also observed in MS and MS fragmentation analysis (Fig. S2, ESI†). However, a major compound with the same UV absorption has emerged in our HPLC analysis, as indicated in our time course assays (Fig. 2(A)). LC-MS and MS fragmentation analysis indicated that this accumulated compound is prelegonmycin A 11 that lacks the hydroxyl group at the C7 position (Fig. S3, ESI†). It is worth noting that there was a large portion of substrate 8 that remained even after extensive reaction time (6 h) (Fig. 2(A)). Although, to the best of our knowledge, there is no literature supporting that recombinant proteins with TF tags affect the overall enzymatic activities,^32^ attempts were made to remove the TF tag from the recombinant LgnC. However, such cleavage resulted in the complete loss of soluble proteins and enzymatic activities (data not shown).

To investigate this further, we cloned the *lgn*C into pGM1190, a self-replicative shuttle vector with a thiostrepton-inducible promoter PtipA used for gene expression in Streptomyces.^33^ The subsequently isolated pGM1190-*lgn*C was directly introduced into *Streptomyces coelicolor* M1152, a heterologous host for recombinant protein production,^34^*via* conjugation for heterologous expression without further modification. The protein extract containing overexpressed recombinant LgnC was used for enzyme assays. Addition of 8 into this protein extract, together with exogenous FAD (0.1 mM) and NADPH (1 mM), resulted in the accumulation of 11 with only a trace amount of 3 in our time course experiments (Fig. 2(B)). It appears that all of 8 was consumed after a 4-h assay as observed in HPLC traces. Interestingly, the NADPH recycling system was no longer required to complete the transformation from 8 to 11. Similar to what was observed in the assays of LgnC-TF, only a trace amount of 3 was produced during these time-course experiments. However, purification of recombinant LgnC from the Streptomyces host proved to be difficult due to the low expression level, often resulting in protein contamination.

Taken together, it is likely that, while the recombinant LgnC is able to efficiently catalyse the conversion of 8 to 11, the final hydroxylation in our *in vitro* assays may result from spontaneous oxidation on the activated C7 position of the electron-rich pyrrole scaffolds, similar to the observation found during the synthesis of legonmycins.^35^

**Fig. 3:**
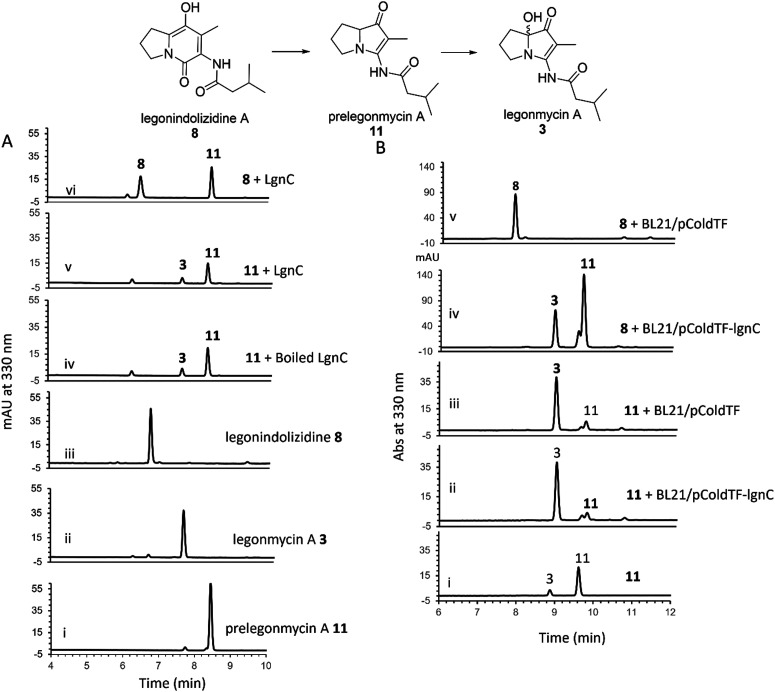
(A) HPLC analysis of the assays containing recombinant LgnC-TF with purified 11. Traces i-iii, standard compounds 11, 3, and 8, respectively. Trace iv. The negative control assay by incubating 11 with boiled LgnC-TF (inactivated). Trace v. The assay by incubating 11 with LgnC-TF. Trace vi. The positive control assay by incubating 8 with LgnC-TF. (B) HPLC analysis of the whole cell assays with either 8 or 11. Trace i, standard compound 11 with a trace amount of 3, possibly coming from spontaneous oxidation. Trace ii. The assay of incubating 11 with whole cells containing overexpressed LgnC-TF. Trace iii. The assay of incubating 11 with whole cells only containing empty plasmid. Trace iv. The assay of incubating 8 with whole cells containing overexpressed LgnC-TF. Trace v. The assay of incubating 8 with whole cells containing an empty plasmid.

To further investigate the hydroxylation, we set out to isolate compound 11*via* large-scale biotransformation and multi-rounds of semi-preparative HPLC separations. MS and MS^2^ fragmentation analysis confirmed the molecular identity of 11, displaying a similar UV profile to the ones of 3 (Fig. S4, ESI†). In a controlled experiment where the boiled recombinant LgnC (inactivated enzyme) was used, 11 remained unchanged with a small amount of 3 presented, which likely originated from the spontaneous oxidation of 11 during this overnight incubation (Fig. 3(A) trace iv). An assay with recombinant LgnC-TF and 8 showed a similar level of 11 produced compared to the control experiment with a small amount of 3 (Fig. 3(A) trace v). This strongly indicated that LgnC is only responsible for the conversion of 8 to 11 (Fig. 3(A), trace vi), but it is unable to install the hydroxyl group on C7 of 11 to provide 3. However, the slow spontaneous oxidation rate on 11 observed in our *in vitro* assays was only able to result in a trace amount of 3 and may not be able to account for our previous observations where only the hydroxylated products were isolated in the original producing isolate, *Streptomyces* sp. MA37 (MA37) and the heterologous expression strain, *S. albus*:7G1.^18^ This suggests that the final hydroxylation at the C7 position in some bacterial PAs results from an endogenous redox reagent present (*i.e.* a promiscuous hydroxylase or even hydrogen peroxide) in the host cells.

To confirm this, we set out to investigate whether we are able to observe efficient hydroxylation on 3 in the *E. coli* whole cell biotransformation. The benefit of cell-based biotransformation lies in the ability to utilize multistep cascade reactions linked to oxidative pathways, as many biochemicals require consecutive enzymatic modifications for effective synthesis and functionality.^36^ Another benefit during the synthesis is that coenzymes can be provided through intracellular metabolism, or reducing potential can be supplied through cofactor generation in cells with high volumetric activity.^36^ To this end, we first utilized *E. coli* BL21(DE3) harbouring the overexpression construct pColdTF-lgnC as the catalyst.^18^ Overnight incubation of compound 8 in the cell-based system led to its complete conversion to compounds 11 and 3, as confirmed by HPLC analysis (Fig. 3(B), trace iv). In a control experiment, where BL21(DE3) cells harboring the empty plasmid (BL21/pCold-TF) were used, no conversion of 8 was observed, indicating that the transformation to 11 and 3 requires recombinant LgnC (Fig. 3(B), trace v). Interestingly, when 11 was added to both the BL21/pCold-TF-LgnC (Fig. 3(B), trace ii) and BL21/pCold-TF (Fig. 3(B), trace iii) systems, it was efficiently converted to compound 3. This demonstrates that the final hydroxylation of 11 to 3 is independent of recombinant LgnC but can be mediated by endogenous redox reagents. The 11-to-3 ratio in the cell-based system was approximately 3 : 1, suggesting that the conversion to 3 is more efficient in the whole-cell system compared to *in vitro* assays.

In contrast, incubation of 8 in cell-free lysates containing overexpressed LgnC-TF resulted primarily in the accumulation of 11, with only trace amounts of 3 (Fig. S5, ESI†). Additionally, no conversion of 8 to 11 or 3 was observed in control lysates from BL21(DE3) containing the empty pColdTF plasmid, in line with previous *in vitro* observations (Fig. 3(A)).

These results suggest that the complete conversion of 8 to 11 is catalyzed by recombinant LgnC, whereas the final hydroxylation step to form 3 is likely driven by an endogenous hydroxylase or hydrogen peroxide produced by *E. coli* BL21(DE3) under aerobic conditions.^37^ The diminished production of hydroxylated products in cell-free lysates may be due to reduced activity of this endogenous redox system.

**Fig. 4:**
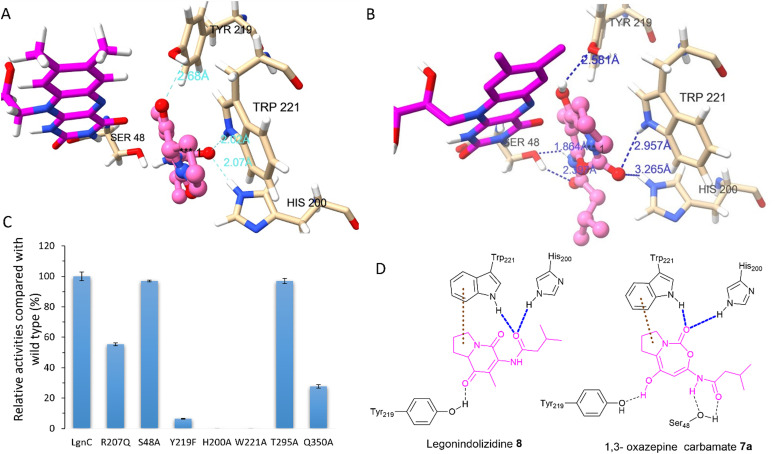
A model of LgnC active site bound with FAD cofactor (pink) was generated by AlphaFold 3. (A) Docked legonindolizidine 8 (salmon) interacting with key residues in the active site of LgnC. (B) Docked 1,3-oxazepine carbamate 7a (salmon) interacting with key residues in the active site of LgnC. (C) The relative enzyme activities of protein variants compared to wild-type LgnC. The biotransformation was measured from legonindolizidine A 8 to prelegonmycin A 11. (D) A plain structural elucidation of the interactions between LgnC key amino acid residues and legonindolizidine 8 (left) and 1,3-oxazepine carbamate 7a (right). Blue dashed lines represent hydrogen bonding interactions and brown dashed lines represent hydrophobic interactions.

Attempts to crystallize recombinant LgnC-TF were made owing to its unusual multistep catalysis. Despite repeated trials, we were unable to obtain a crystal structure of recombinant LgnC-TF. Nonetheless, we sought to understand the ability of LgnC to have a bound FAD cofactor by using the recently developed AlphaFold 3 algorithm.^38^ The predicted structure of LgnC with a bound FAD cofactor was then submitted to the Dali server^39^ for 3D structural comparison. The predicted LgnC structure displays 26% structural similarity (*Z*-scores of 35.2, rmsd value of 2.8) to AbsH3, an FAD-dependent reductase from the abyssomicin biosynthetic pathway,^40^ the highest %id among other structures of FAD-dependent oxidoreductases. Similar to AbsH3, LgnC appears to have two “domains” (Fig. S6, ESI†), site A containing residues 5–73, 90–176, 270–345, and 370–381, and site B containing residues 70–90, 177–269 and 346–369 (Fig. S6, ESI†). The arrangement of bound FAD in site A is similar to AbsH3 (Fig. S7, ESI†). One cavity was predicted by CAVER software^41^ in close proximity to the quinone of the FAD cofactor (Fig. S8, ESI†). This void appears to link channels to facilitate the transport of substrates and products in site B (Fig. S8, ESI†). We then conducted docking studies on legonindolizidine A 8 and the transient 5+7 7a to gain insights into how LgnC handles the substrate and intermediate.^42^ In substrate 8, the hydrophobic 5+6 ring system appears to be inserted into a pocket between the indole ring of W221 and the quinone moiety of the FAD cofactor through hydrophobic interaction with W221 (Fig. 4(A)). The oxygen amide at the isovaleryl motif of 8 also engages in hydrogen bonding interactions with H200 and NH of W221 (Fig. 4(A)). Such an insertion of 8 between FAD and W221 is likely to be further supported by the hydrogen bonding interactions between the oxygen at C1 of 8 and the hydroxyl group of Y219 (Fig. 4(A)). While the overall position of 1,3 oxazepine carbamate intermediate 7a remains similar to 8, and Y219 also forms H-bonding with the oxygen at C1 of 7a, but there was variability observed in the docking results in the initial trajectory and binding position of 7a (Fig. 4(B)). This variability could account for the ring expansion from the original 8 to the transient 1,3-oxazepine carbamate intermediate 7a. We observed that, while the amide oxygen of the isovaleryl motif is likely to form the H-bonding interaction with S48, NH of W221 and H200 instead forms the H-bonding interactions of the carbonyl oxygen of the carbamate motif (Fig. 4(D)). This suggests that NHs of H200 and W221 may form an oxyanion hole to stabilise the transient intermediate after the nucleophilic attack initiated by the FAD-C4a-hydroperoxide ion for the subsequent structural rearrangement (Fig. S9, ESI†).

Interestingly, we also observed that residues R207, T295, and Q350 are in close proximity to the exit tunnel and within 6 Å distance of the H200 and W221 (Fig. S8D, ESI†). Such hydrophilic surroundings may position one water molecule close to the carbonyl carbon of 1,3 oxazepine carbamate intermediate 7a to facilitate the ring opening, followed by the final decarboxylative ring contraction.

To investigate the catalytic roles of the above identified residues, we performed site-directed mutagenesis (SDM). Changing His200 and Trp221 to Ala, resulting in protein variants H200A and W221A (Fig. S10, ESI†), respectively, completely abolished the enzymatic activities of LgnC from 8 to 11 (Fig. 4(C)). The protein variant, Y219F, lost 95% activity compared to the wild-type (WT) LgnC. While these three residues are absent in other BVMOs, they are highly conserved in other LgnC BVMO homologues (Fig. S11, ESI†), strongly suggesting that they indeed are key to the biotransformation of oxygen insertion from [5+6] indolizidines to [5+7] 1,3 oxazepine carbamate intermediates. While protein variants S48A and T295A (Fig. S10, ESI†) retained almost the same activity as the WT LgnC, the enzyme activities were reduced to approximately 50% and 30% compared to the WT LgnC in the assays of protein variants R207Q and Q350A (Fig. S10, ESI†), respectively. Partial loss of enzymatic activities in protein variants, R207Q and Q350A, suggested that these residues may have some impact on the enzyme activity (possibly water activation).

In conclusion, we characterized the enzyme activity of the BVMO, LgnC, that catalyses the conversion of legonindolizidine A 8 to prelegonmycin A 11, the last intermediate in the biosynthesis of legonmycin A. The final hydroxylation at the C7 position of 11 is likely to originate from either spontaneous oxidation by hydrogen peroxide or an endogenous hydroxylase in bacterial strains. Structural modelling of LgnC with FAD cofactor and subsequent substrate docking enabled the identification of key amino acid residues in the putative active sites. Our SDM experiments revealed that Y219F, H200A, and W221A play important roles in biotransformation, which are likely to position legonindolizidine A 8 to facilitate the proposed Baeyer–Villiger ring expansion. Further insights into the molecular mechanism will require protein structures of LgnC or its homologues and the protein complexes with the corresponding substrates.

## Materials and methods

### Protein expression and purification

The pCold-TF-lgnC plasmid and its derivatives were individually transformed into *E. coli* BL21 (DE3). Single colonies from each transformation were grown overnight in LB media (5 mL) containing ampicillin (50 μg mL^−1^). The overnight culture was transferred to fresh LB medium (500 mL) supplemented with ampicillin (50 μg mL^−1^) and cultivated at 37 °C until the cell density reached an OD_600_ of 0.6. IPTG was added to a final concentration of 0.1 mM to induce protein expression. Cells were grown for 16–20 h at 16 °C and then harvested by centrifugation at 4 °C. The cell pellets were resuspended in ice-cold lysis buffer (20 mM tris–HCl, 300 mM NaCl, 10 mM imidazole, pH 8.0), and further disrupted by an Ultrasonic Homogenizer JY92-IIN. Then, the supernatant of cell debris was loaded onto the Ni-NTA-affinity column. Bound proteins were eluted with the same tris–HCl buffer containing different concentrations of imidazole. The desired elution fractions were combined and concentrated using a Centrifugal Filter Unit (Millipore). The purified proteins were stored at −80 °C in storage buffer (100 mM Tris–HCl, pH 8.0, 150 mM NaCl, 10% (w/v) glycerol, 1 mM DTT). The protein concentrations were determined on a NanoDrop 2000 by using the corresponding extinction coefficient.

### Activity assay for LgnC and its mutants

Activity of LgnC and its mutant was assayed by monitoring the conversion of substrates into products as analyzed by HPLC. Assays were set up containing 10 μM purified protein, 0.3 mM substrate, 0.1 mM FAD, 1 mM NADPH, and 15 μL NADPH recycling system^18^ in a final volume of 50 μL. After incubating at 28 °C over time, the reactions were quenched by the addition of two equal volumes of methanol, and protein precipitates were removed by centrifugation. The supernatants were then analyzed using an Agilent Technologies 1260 infinity HPLC system equipped with an ACE C18 10 μm 10 × 250 mm column and PDA detector monitoring absorbance at 330 nm. Samples were separated on a linear gradient of 5% to 80% methanol (v/v) in water over 20 min at a flow rate of 1 mL min^−1^.

### Streptomyces cell-free assay for LgnC

*S. coelicolor* M1152/pGM1190-lgnC was inoculated into 50 mL of 10.3% YEME with 10 μg mL^−1^ apramycin and cultivated at 30 °C and 180 rpm for 2 days. 50 μL of 25 μg mL^−1^ thiostrepton was then supplemented into the culture to induce the expression of LgnC. After 3-day fermentation, 2 mL of cell culture was collected by centrifugation, resuspended in 800 μL tris–HCl buffer (50 mM tris–HCl, 125 mM NaCl, pH 8.0), and lysed with sonication on ice. The cellular debris was removed from the lysate by centrifugation. A typical 50-μL assay solution contained 40 μL of the above cell-free extract, 0.3 mM substrate, 1 mM NADPH and 0.1 mM FAD. The reactions were performed at 28 °C during the time course experiments. At each time point, the reaction was quenched by the addition of two equal volumes of methanol, and protein precipitates were removed by centrifugation prior to HPLC detection.

### Preparation of prelegonmycin A

Large scale reaction using M1152-lgnC cell-free extract was conducted to obtain a sufficient amount of prelegonmycin A. To initiate the reaction, legonindolizidine (1.7 mg) was added to 20 mL of freshly prepared M1152-lgnC cell-free extract supplemented with 0.5 mM NADPH and 50 μM FAD. After incubating at 28 °C for about 16 h, the reaction was terminated by the addition of an equal volume of methanol, and the protein precipitate was removed by centrifugation. The supernatant was dried and redissolved in methanol. The crude extract was then purified by reverse-phase HPLC (ACE 5 μm, 250 × 10 mm) on a linear gradient of 40% to 76% methanol over 18 min at a flow rate of 2 mL min^−1^, to give prelegonmycin A (*t*_R_ = 14.7 min, 1.2 mg).

### Whole cell reaction assay for LgnC

1 mL overnight culture of BL21/pCold-TF-lgnC was transferred into 50 mL SOB with 50 μg mL^−1^ Amp and cultivated at 37 °C, 180 rpm. When the OD_600_ of the medium reached 0.6, IPTG was added to a final concentration of 0.1 mM, and the culture was further incubated at 16 °C for 18 h. 2 mL of the culture was collected by centrifugation for each reaction and washed with 1 mL of potassium phosphate buffer (100 mM, pH 7.4) three times. The washed cells were resuspended in 500 μL of potassium phosphate buffer and supplemented with legonindolizidine prior to the incubation at 28 °C. At the end of the incubation period, cells were removed by centrifugation. The supernatant was extracted twice with ethyl acetate, the combined organic layers of which were dried and dissolved in 200 μL of methanol.

### Site-directed mutagenesis of recombinant LgnC-TF

Site-directed mutagenesis of LgnC-TF was performed using an In-Fusion® HD Cloning kit (Takara Bio). PCR primer couples containing the desired mutant site were designed to amplify two DNA fragments that overlap each other by 15–20 bp from the template pColdTF-lgnC, respectively (see ESI,† Table S1 for primer sequences), and cloned into the BamHI/HindIII site of pET-28a (+) vector. All constructed plasmids were verified by DNA sequencing.

### HR-MS analysis

High-resolution electrospray ionisation mass spectrometry (HR-ESIMS) was determined using LC MS Thermo Scientific MS system (LTQ Orbitrap) coupled to a Thermo Instrument HPLC system (Accela PDA detector, Accela PDA autosampler, and Accela Pump, C18 Sunfire 150 × 46 mm Waters®). The following parameters were used: capillary voltage 45 V, capillary temperature 320 °C, auxiliary gas flow rate 10–20 arbitrary units, sheath gas flow rate 40–50 arbitrary units, spray voltage 4.5 kV, mass range 100–2000 amu (maximum resolution 30 000). Additionally, the samples were run on an Agilent 6200 series TOF/6500 series Q-TOF instrument with a scan rate of 1.5 Hz, scan range from 100–1000, variable CID energy, 3.5 kV source voltage, fragmentor 175 V (±200%) and reference masses (121.05087 and 922.00979) enabled. The instruments were optimised as required.

### LgnC modelling and substrate docking

The protein sequence of LgnC (UniPort: A0A0U1XUB1) was supplied to the AlphaFold 3 server to generate a structural prediction bound with one molecule of FAD cofactor using the default parameters. Legonindolizidine A and 1,3 oxazepine carbamate models were generated from SMILES strings at the CACTUS server (https://cactus.nci.nih.gov/translate/). Substrate/intermediate and protein models were prepared for docking and used in docking experiments with AutoDock Vina^43^ using the default parameters.

## Data availability

Data for this manuscript is available within the text or the ESI.†

## Conflicts of interest

There are no conflicts to declare.

## Supplementary Material

CB-OLF-D4CB00186A-s001
