# Supplementary material for: Characterization of the Baeyer–Villiger monooxygenase in the pathway of the bacterial pyrrolizidine alkaloids, legonmycins
Source: RSC Chem Biol. 2024 Sep 30;5(11):1177–85. doi: 10.1039/d4cb00186a (PMC11457151; doi:10.1039/d4cb00186a)
Supplement: CB-005-D4CB00186A-s001 [file CB-005-D4CB00186A-s001.pdf]

Supplementary Information (SI) for RSC Chemical Biology.
This journal is © The Royal Society of Chemistry 2024

Characterization of the Baeyer-Villiger monooxygenase in the pathway of the bacterial pyrrolizidine alkaloids, legonmycins

Shan Wang [1,2*], Fleurdeliz Maglangit[3], Qing Fang[2], Kwaku Kyeremeh[4], Hai Deng[2*]

1. State Key Laboratory of Microbial Technology, Shandong University, Qingdao 266237, China.
2. Department of Chemistry, School of Natural and Computing Sciences, University of Aberdeen, Aberdeen AB24 3UE, United Kingdom.
3. Department of Biology and Environmental Science, College of Science, University of the Philippines Cebu, Lahug, Cebu City 6000, Philippines
4. Marine and Plant Research Laboratory of Ghana, Department of Chemistry, University of Ghana, P.O. Box LG56, Legon-Accra, Ghana

*Corresponding authors:

Shan Wang, email: shan.wang@sdu.edu.cn

Hai Deng, email: h.deng@abdn.ac.uk

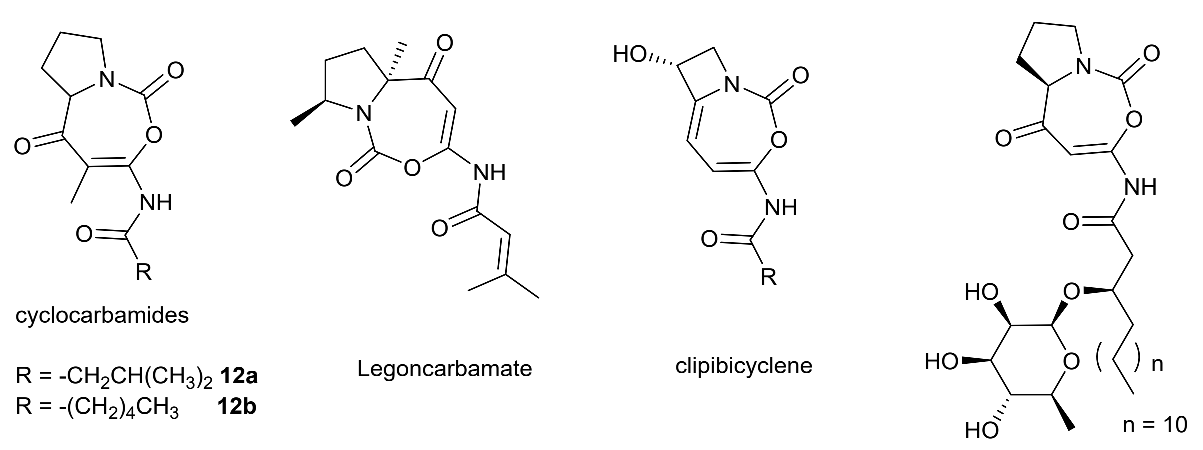

cyclocarbamides

R = -$CH_2CH(CH_3)_2$ **12a**
R = -$(CH_2)_4CH_3$ **12b**

Legoncarbamate

clipibicyclene

n = 10

SB-315021

Figure S1. Representative structures of bacterial cyclocarbamides and their structural homologues[26,28-30].

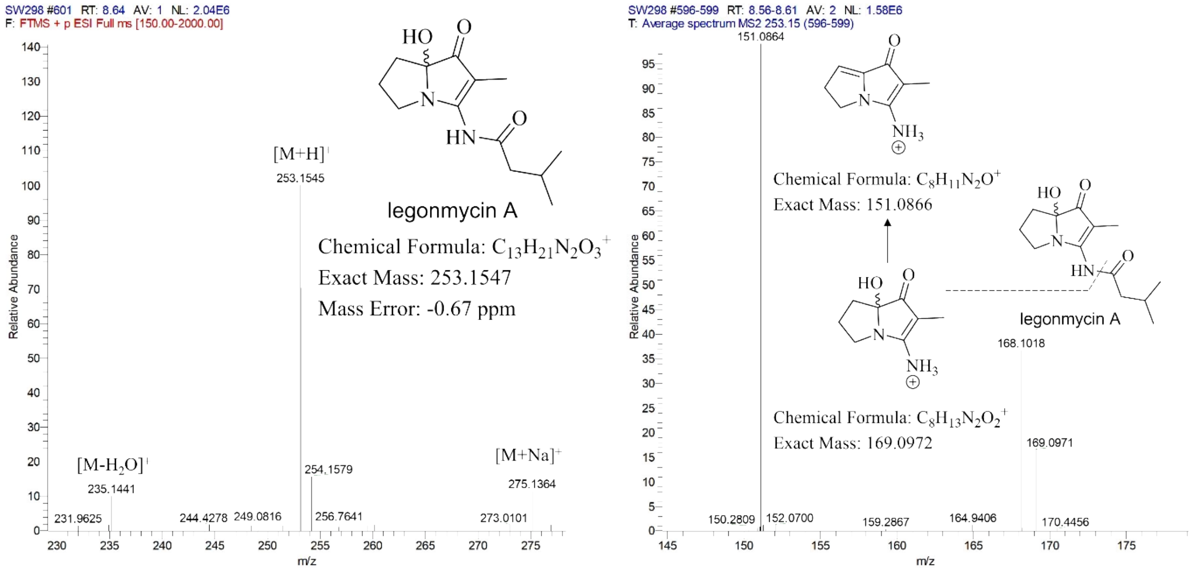

Figure S2. MS and MS^2 fragmentation analysis of legonmycin A **3**.

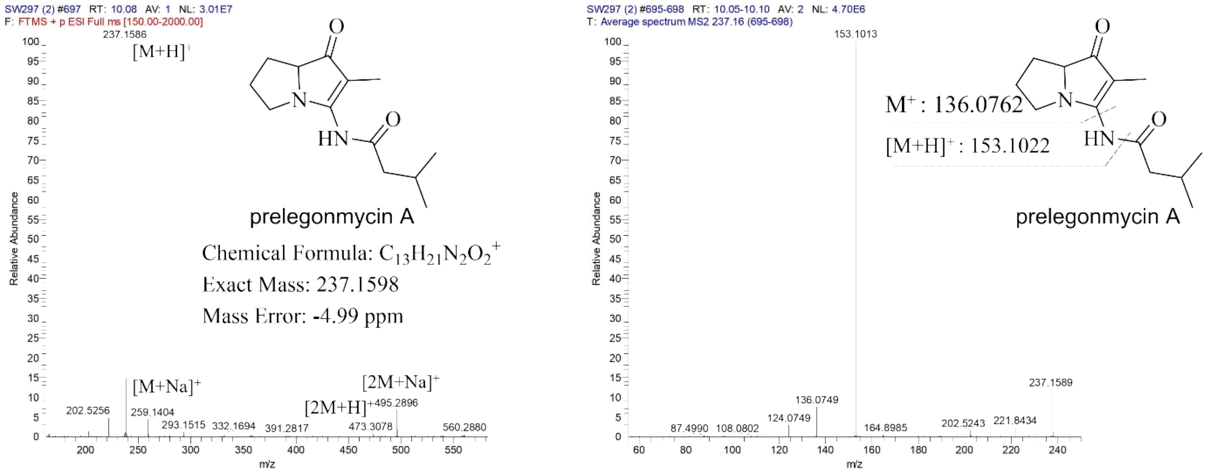

Figure S3. MS and MS^2 fragmentation analysis of prelegonmycin A **11**.

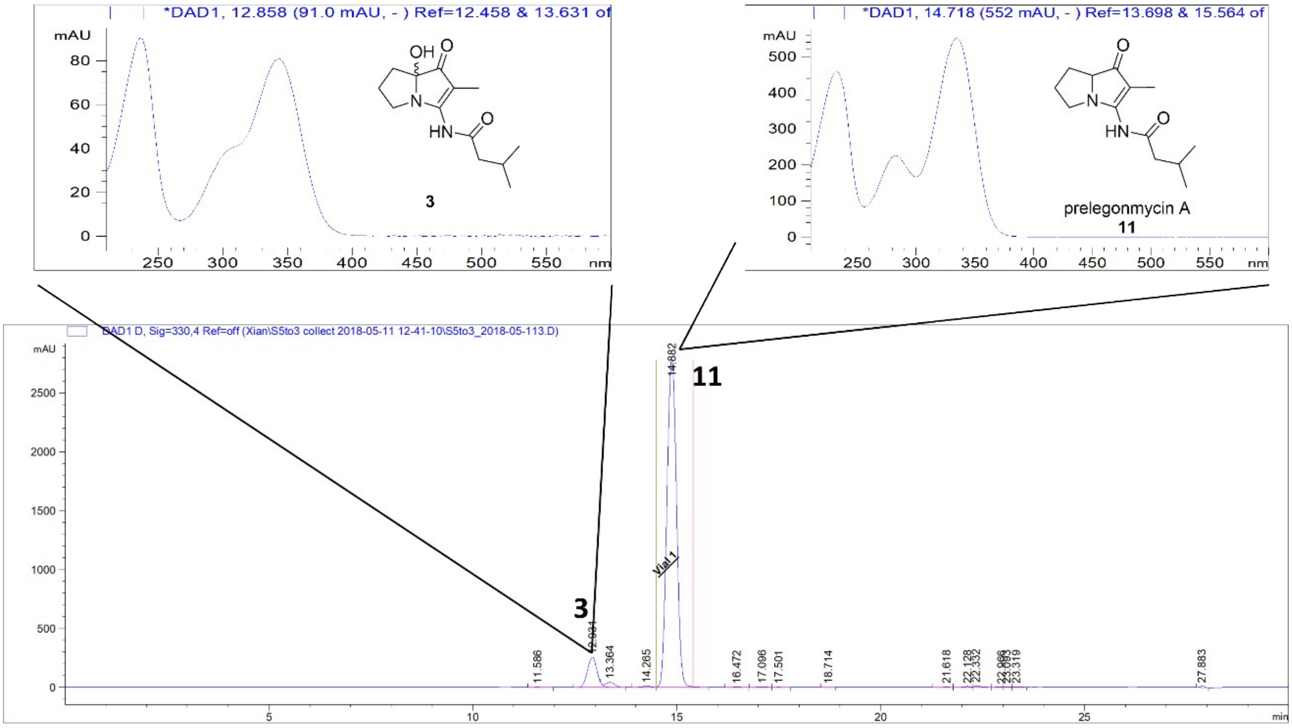

Figure S4. Semi-preparative separation of prelegonmycin A **11** from the large-scale biotransformation of recombinant LgnC and legonindolizidine A **8** with all of necessary co-factors. Top left. UV absorption of legonmycin A **3**. Top right. UV absorption of prelegonmycin A **11**. Bottom. HPLC traces of the biotransformation.

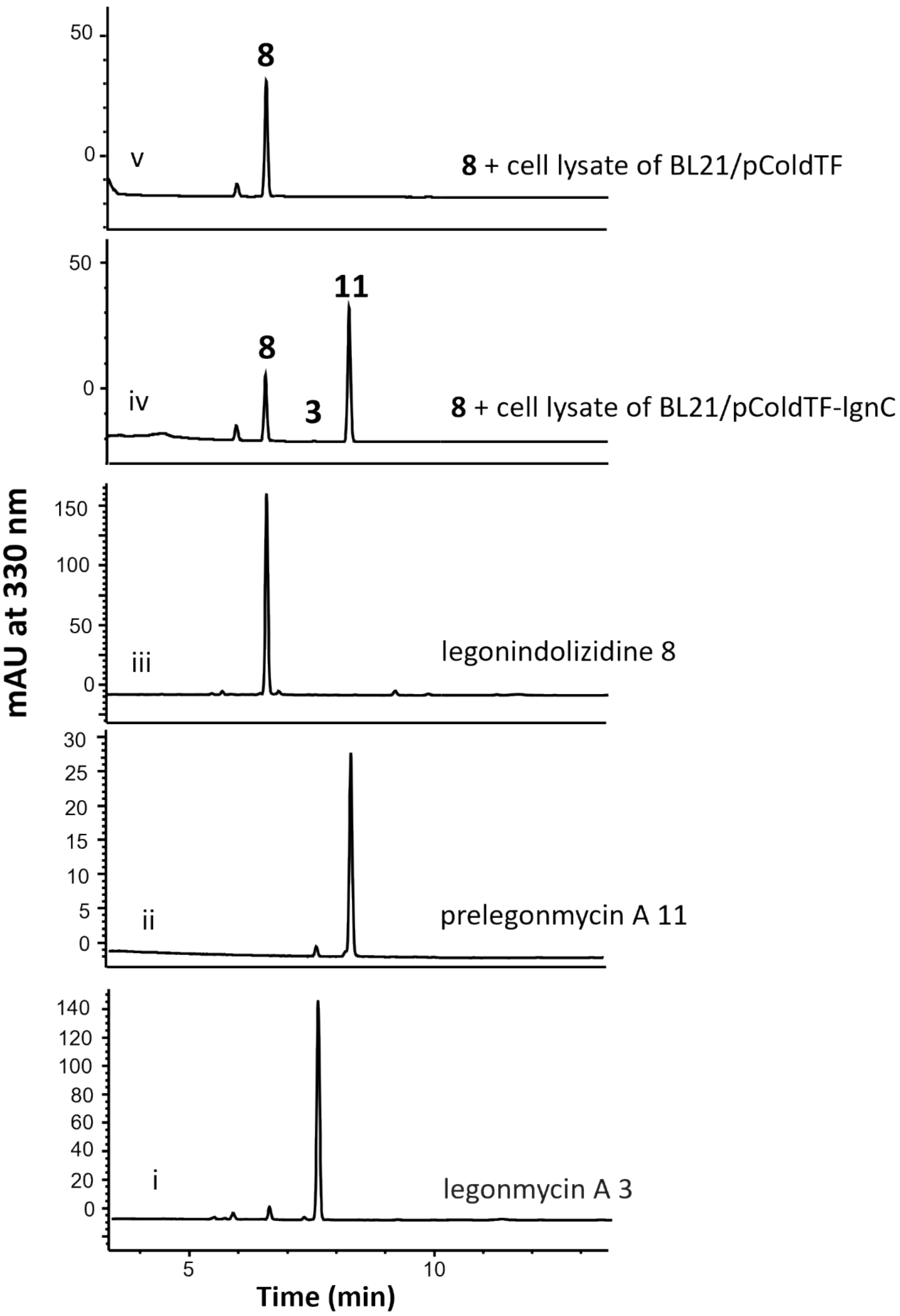

Figure S5. HPLC traces of the biotransformation from **8** to **11** and **3** using the cell-free lysate of BL21(DE) harbouring pColdTF-lgnC. **A**. Trace i. standard legonmycin A **3**; ii. the standard **11**; iii. the standard **8**; vi. The overnight assays showing the conversion of **8** to **11** with a trace amount of **3**; v. the control experiment using the cell-free lysate of BL21(DE) harbouring empty pColdTF.

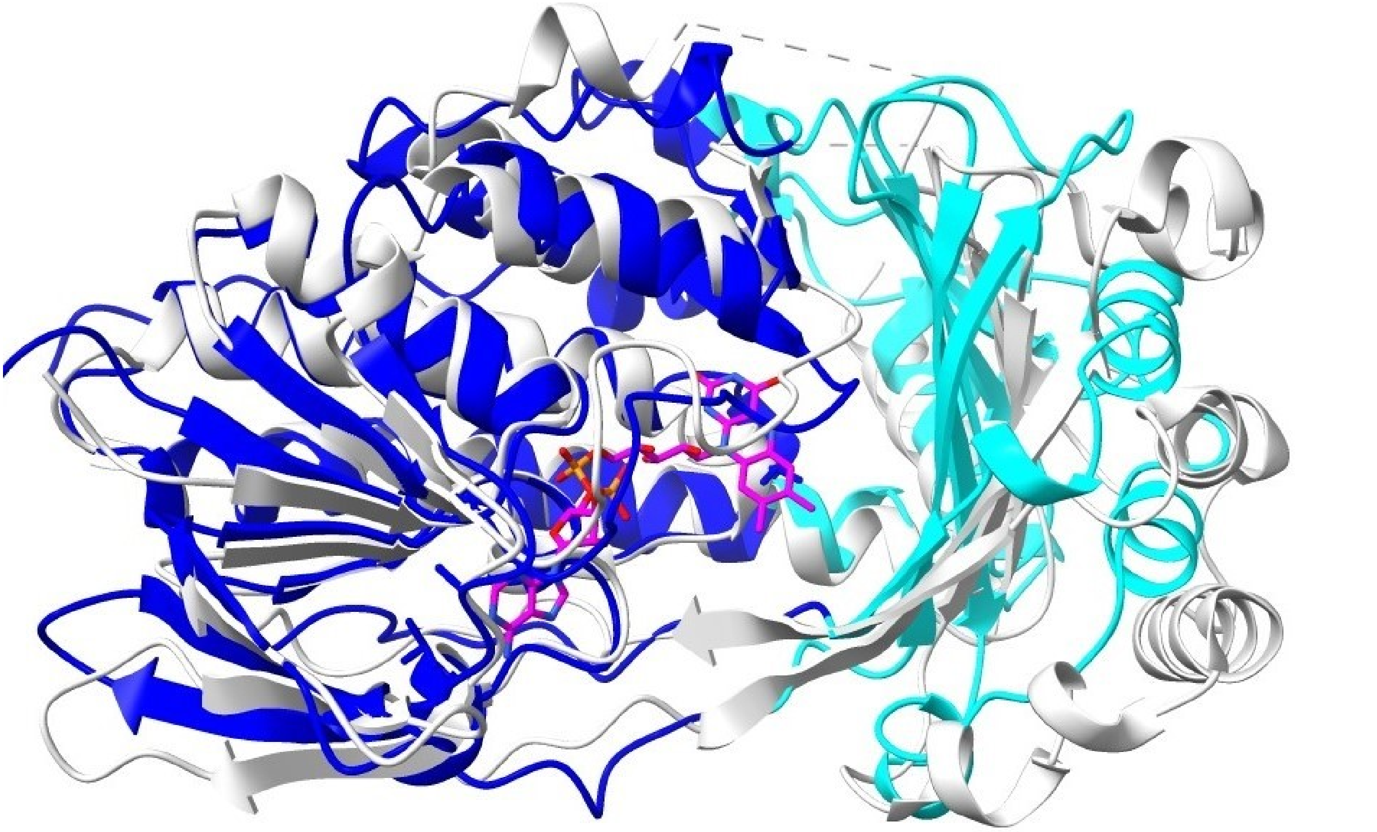

Figure S6. Overlapped structural comparison of the FAD-dependent reductase, AbsH3 (light grey), and LgnC model bound with FAD (pink) was generated by AlphaFold 3, indicating two domains (blue and cyan).

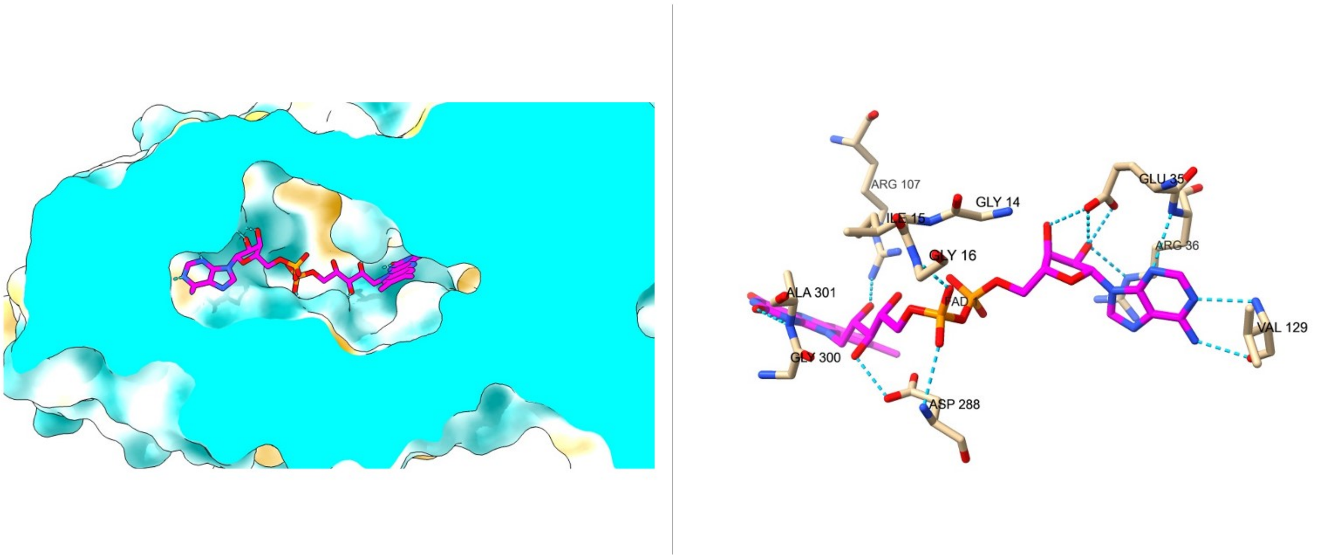

Figure S7. FAD binding sites in the model of LgnC generated by AlphaFold 3. Left. The cavity of FAD binding sites. Right. Key amino acid residues that have H-bonding interactions with FAD molecules.

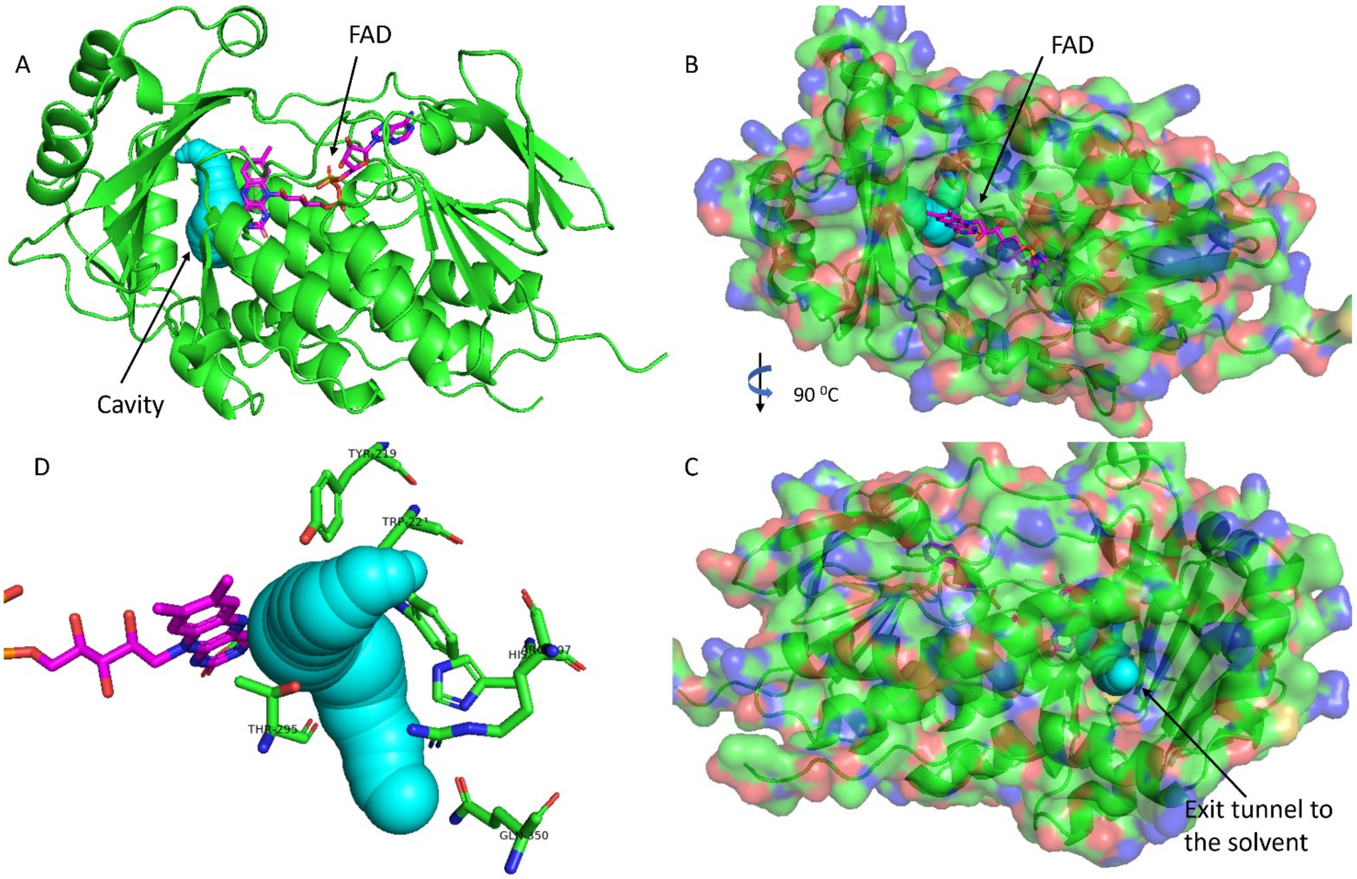

Figure S8. **A**. One cavity (cyan tunnel) was predicted by CAVER software in close proximity to the quinone of the FAD cofactor (pink). **B**. One side of LgnC surface shows the binding site of FAD links to the identified cavity (cyan tunnel). **C**. the side of LgnC surface (90 °C turn) shows an exit channel to the solvent. **D**. key residues identified in the close proximity of the identified cavity (cyan tunnel). Among these residues, Arg207, Thr295, and Gln350 appear to be located in the exit channel, and Ser48, His200, Tyr219, and Trp221 are in the centre of the cavity that may assist the binding of legonindolizidine and 1,3-oxazepine carbamate.

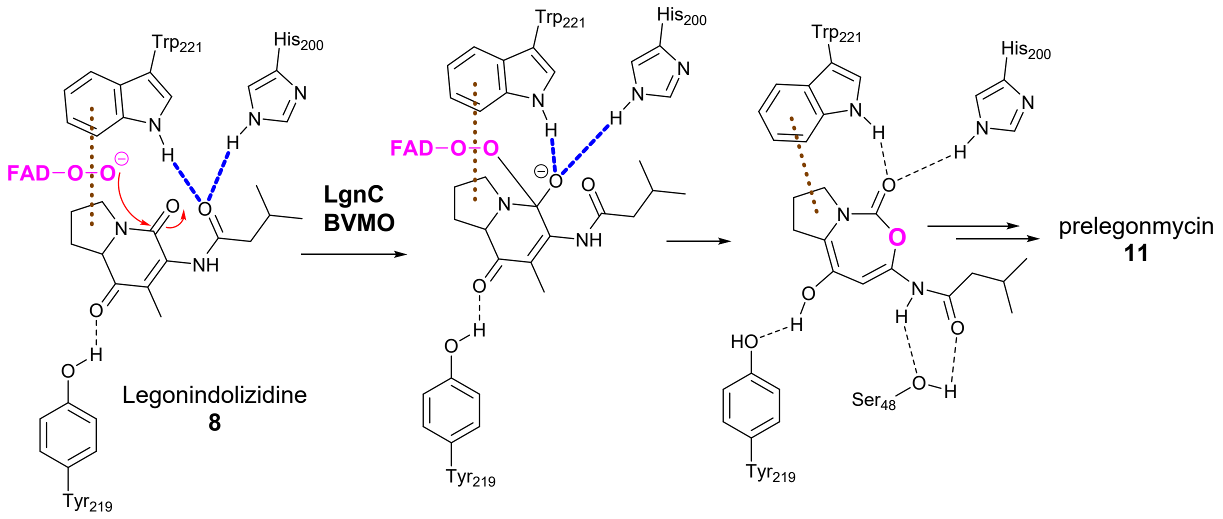

Figure S9. A proposed mechanism of the biotransformation from 8 to 11 catalysed by LgnC. Blue dash lines represent hydrogen bonding interactions and brown dash lines represent hydrophobic interactions.

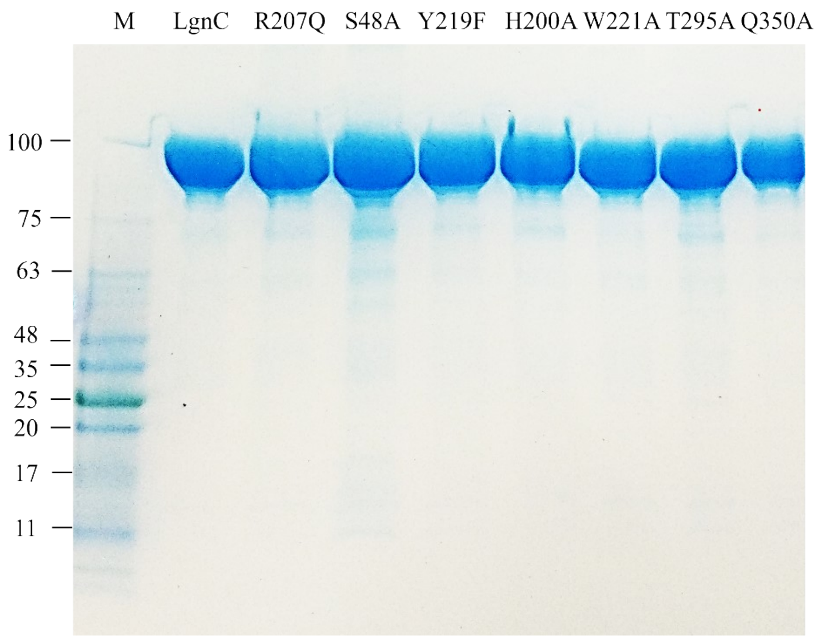

Figure S10. SDS-PAGE analysis of purified LgnC-TF and its mutant.

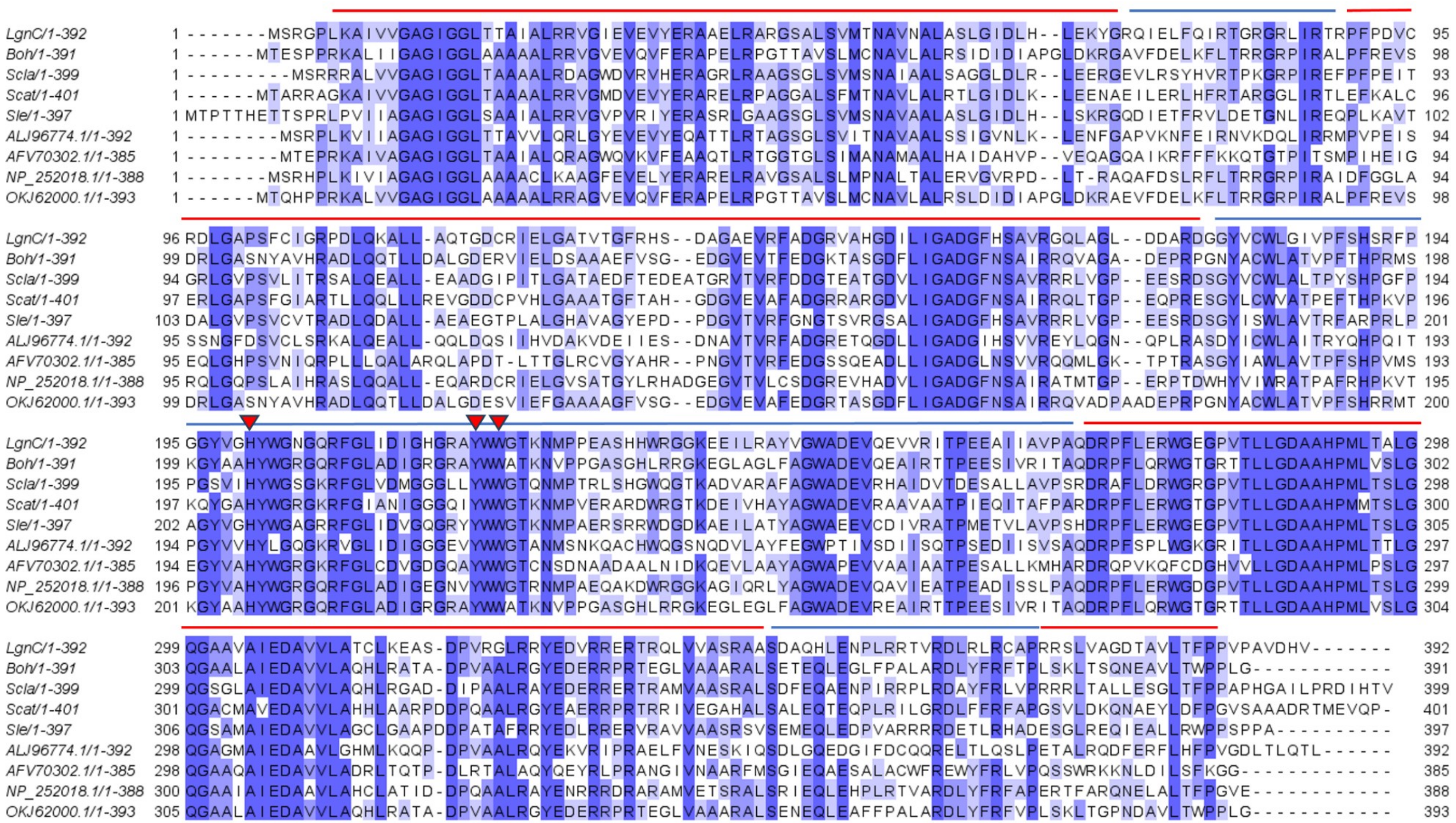

Figure S11. Protein alignment of LgnC and other LgnC-like BVMOs suggests the presence of three highly conserved residues, His200, Tyr219 and Trp221 (LgnC number, highlighted in red triangle) that play important roles in the BV biotransformation. The red line above the AA sequence belongs to 'site A' while the blue belongs to 'site B'.

Table S1. Primers used in this study.

Primer	Sequence	Use
LgnC-SM-For1	TCGGTACCCTCGAGGGATCCATGAGCCGT	Construction of LgnC mutants
LgnC-SM-Rev2	GACTGCAGGTCGACAAGCTTTTACACATGGTCAAC	
LgnC-RtoQ-F2	CAACAGTTTGGCCTGATCGATATTGGTCACG	Construction of LgnC (R207Q)
LgnC-RtoQ-R1	TCGATCAGGCCAAACTGTTGGCCGTTACCCC	
S48toA-rev1	GTTAACCGCGTTGGTCATCACCGCCAGC	Construction of LgnC (S48A)
S48toA-for2	GTAGCGCGCTGGCGGTGATGACCAAC	
Y219toF-for2	GGTCACGGCCGTGCGTTCTGGTGGGGTA	Construction of LgnC (Y219F)
Y219toF-rev1	ATGTTCTTGGTACCCCACCAGAACGCACGG	
H200toA-for2	CTACGTTGGTGCGTATTGGGGTAACG	Construction of LgnC (H200A)
H200toA-rev1	CAATACGCACCAACGTAGCCACCCGGAA	
W221toA-for2	GGTCACGGCCGTGCGTACTGGGCGGGTA	Construction of LgnC (W221A)
W221toA -rev1	ATGTTCTTGGTACCCGCCCAGTACGCACGG	
T295toA-for2	GATGCTGGCGGCGCTGGGTCAAGGTGCT	Construction of LgnC (T295A)
T295toA-rev1	GACCCAGCGCCGCCAGCATCGGGTGC	
Q350toA-for2	CGACGCGGCGCACCTGGAAAACCC	Construction of LgnC (Q350A)
Q350toA-rev1	TTTCCAGGTGCGCCGCGTCGCTCG	
